# Motor outcomes in individuals born small for gestational age at term: a systematic review

**DOI:** 10.1186/s12887-024-05187-y

**Published:** 2024-11-11

**Authors:** Hoda Taiar, Silje Dahl Benum, Kristina Anna Djupvik Aakvik, Kari Anne I. Evensen

**Affiliations:** 1 Faculty of Medicine and Health Sciences, Norwegian University of Science and Technology, Trondheim, Norway; 2 Department of Clinical and Molecular Medicine, Norwegian University of Science and Technology, Trondheim, Norway; 3 Children’s Clinic, St. Olavs Hospital, Trondheim University Hospital, Trondheim, Norway; 4 Department of Rehabilitation Science and Health Technology, Oslo Metropolitan University, Oslo, Norway

**Keywords:** Fetal growth restrction, Intrauterine growth restriction, Motor skills, Small for gestational age, Systematic review

## Abstract

**Background:**

Being born small for gestational age (SGA) is a risk factor for motor difficulties. Previous reviews exploring this topic are mostly focused on children born preterm. We aimed to review the literature to determine the association between being born SGA at term and motor outcomes.

**Methods:**

PubMed and Embase were searched for relevant articles without any restrictions on publication year or participants’ age. Inclusion criteria were SGA exposure at term (≥ 37 weeks of gestation), cohort studies or randomized controlled trials with motor outcome assessed by standardized motor tests with results reported as continuous scores (mean/median) compared with a control group. Exclusion criteria were abstracts, editorials and commentaries, articles in non-English language or no full text available. Reviews were screened for relevant articles. Quality of included studies was assessed by the Newcastle-Ottawa Scale.

**Results:**

In total, 674 records were identified by the literature search and screened by two independent authors. Thirteen original articles were eligible and included in a qualitative synthesis, and five (38%) of these were included in a meta-analysis. Nine (69%) studies were from high-income countries. Most studies were carried out in early childhood, and only one study in adulthood. Seven (54%) articles reported that individuals born SGA at term had poorer scores on standardized motor tests compared with controls, while no differences were reported in five (38%) articles. One article did not report p-values, although the differences were comparable to the other studies. Group differences were of small to moderate effect size (0.19 to 0.65 standard deviation units). The pooled effect size was -0.43 (95% confidence interval: -0.60 to -0.25). Adjustment for covariates were reported in seven (54%) articles and did not change the results. Proportions of motor difficulties, reported in five (38%) articles, ranged from 8.9 to 50% in individuals born SGA from infancy to adolescence.

**Conclusions:**

This systematic review shows that being born SGA, also at term, may be a risk factor for poorer motor outcomes throughout childhood, confirmed by a meta-analysis in early childhood. Further research is needed to establish the risk of adult motor difficulties in individuals born SGA at term.

**Supplementary Information:**

The online version contains supplementary material available at 10.1186/s12887-024-05187-y.

## Background

Individuals born small for gestational age (SGA) are often defined as having a birth weight below the 10th percentile for their gestational age. SGA is the most frequently used indicator of intrauterine growth restriction (IUGR) or fetal growth restriction (FGR), which is a state where the fetus does not reach its genetic growth potential [1]. However, not all infants born SGA are growth restricted. The term SGA includes infants with FGR as well as constitutionally small but healthy infants. FGR implies that the fetus reduces its overall size to increase survival chance as a compensational mechanism against compromised nutrient supply [2], which may be caused by maternal, placental, fetal or genetic factors [3]. If the growth restriction occurs in the third trimester, as is most common, the head circumference and length are spared, resulting in an asymmetric growth restriction with relatively larger reduction in weight than length [4]. Even though the SGA definition is based on birth weight without considering growth *in utero* [3], both FGR and SGA have been associated with long term morbidity varying from physical growth impairment to neurodevelopmental impairment and susceptibility for later diseases, such as metabolic syndrome and cardiovascular diseases [4].

Motor skills play an important role in the daily life of children and adolescents. Motor difficulties are common in children born preterm [5, 6], especially those born very preterm or with very low birth weight [6–9], and may coincide with difficulties in cognition, behavior and academic performance [10–12]. Neurodevelopmental outcomes in children born SGA at term have been less studied than in children born preterm. A few systematic reviews have been published [13–15]. Being born SGA or with IUGR was associated with lower neurodevelopmental scores during childhood [13, 14]. Children born SGA with circulatory redistribution was at even higher risk of neurodevelopmental problems [14, 15]. However, these reviews did not only include children born SGA at term or focus on motor skills. In this systematic review, we synthesized results from studies that investigated motor outcomes in children and adults born SGA at term compared with controls and confirmed the findings by a meta-analysis for a subset of the studies. Our primary aim was to compare scores on standardized motor tests, preferentially controlling for covariates and potential confounders. Our secondary aim was to compare proportions of motor difficulties.

## Methods

### Search strategy

A comprehensive literature search for studies investigating motor outcomes in individuals born SGA at term was carried out by one author (HT) in Medline (PubMed) and Embase in September-October 2023, combining three concepts of interest: ‘SGA’, ‘term-born’ and ‘motor outcomes’. Search terms are listed in Table S1. No limitation regarding publication year was set in our query. We followed guidelines for reviewing retrieved references for inclusion in systematic reviews using EndNote by Bramer et al. [16].

### Study selection

Screening was carried out by two authors (HT and KAIE) using the following inclusion criteria: SGA exposure at term (≥ 37 weeks of gestation) and motor outcome assessed by standardized motor tests with results reported as continuous scores (mean/median) compared with a control group born appropriate for gestational age (AGA) at term. We included only articles that reported original research from cohort studies or randomized controlled trials and were available in full text in English. We excluded abstracts, editorials and commentaries.

**Fig. 1:**
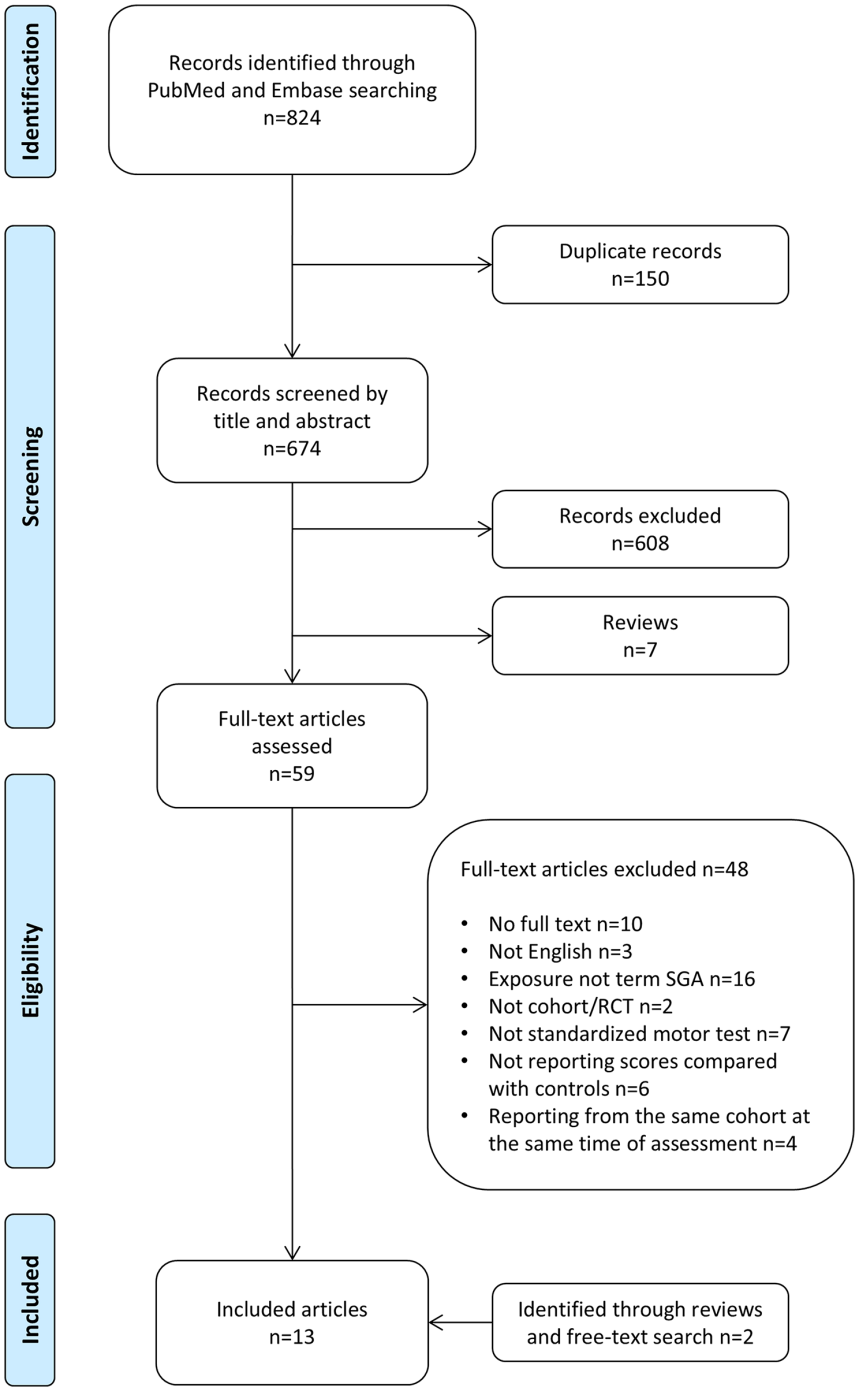
Flow of article selection for review. SGA: Small for gestational age, RCT: Randomized controlled trial

The search produced 824 records, whereof 150 were duplicate records (Fig. 1). After screening of titles and abstracts, the remaining articles were assessed for eligibility by checking the full text against above-mentioned criteria. If results from the same cohort at the same time of assessment were reported in more than one article, the article with the larger sample was included in the review. The full-text assessment resulted in 11 articles.

Seven reviews were identified in the screening process and two more in the full-text assessment. Three had no full text available, two were not in English and two did not include term SGA as exposure. We reviewed the references of the remaining reviews and identified one additional original research article [17]. We additionally performed a free-text search using “neurodevelopmental outcome” and “small for gestational age” identifying one more article [18]. Key characteristics of included original articles were entered into Table 1.

### Quality assessment

The quality of the included studies in terms of selection, comparability and outcome was independently assessed by two authors (SDB and KADA) by using the Ottawa-Newcastle Scale for cohort studies [19]. Assessment criteria were set for each domain (Table S2), and assessment discrepancies were resolved by discussion with a senior author (KAIE).

### Synthesis of results

A qualitative synthesis of results from the included articles was performed. We included mean (standard deviation [SD] or 95% confidence interval [CI]), median (interquartile range [IQR] or range) and p-values as reported in the original articles. P-values below 0.05 were considered statistically significant. We calculated effect sizes in SD units, and magnitude of the difference between groups were interpreted as small (0.2), medium (0.5) or large (0.8) [20]. Five articles reported mean and SD for the SGA and control groups using a version of the Bayley Scales of Infant Development (BSID) in unique study samples between 1 and 2 years of age, allowing us to perform a meta-analysis by random-effects model using IBM SPSS Statistics 29. An overall effect size for BSID was computed weighting each study’s effect size by the study’s sample size. To test heterogeneity of the effect sizes, Q and I^2^ tests were conducted. We grouped covariates into pregnancy factors, birth and neonatal factors, family and socioeconomic factors, child factors at assessment, sex and age. Where proportions of motor difficulties were given, we presented the results in a graph.

## Results

### Background characteristics

Table 1 shows the characteristics of the 13 included articles, published between 2002 and 2023. The studies were from high-income countries in Europe (*n* = 8), USA (*n* = 1) and middle-income countries Brazil (*n* = 3) and India (*n* = 1), and all were cohort studies. Most studies defined SGA as a birth weight below the 10th percentile. Birth year of participants was 1986 to 2019 and mean age at assessment was 40 weeks to 26 years. Several articles reported results from more than one test and/or for more than one timepoint [17, 21–24]. The following motor tests were used (Table S3): Alberta Infant Motor Scale (AIMS) [25] (*n* = 1), BSID [26] (*n* = 2), Bayley Scales of Infant Development – Second edition (BSID-II) [27] (*n* = 2), Bayley Scales of Infant and Toddler Development – Third Edition (BSID-III) [28] (*n* = 3), General Movements Assessment Motor Optimality Score – Revised (GMA MOS-R) [29] (*n* = 1), Grooved Pegboard (GP) [30] (*n* = 2), Hammersmith Infant Neurological Examination (HINE) [31] (*n* = 1), Movement Assessment Battery for Children (Movement ABC) [32] (*n* = 2), Neonatal Behavioral Assessment Scale (NBAS) [33] (*n* = 1), Peabody Developmental Motor Scales (PDMS) [34] (*n* = 2), Test of Infant Motor Performance (TIMP) [35] (*n* = 1), Touwen neurological examination [36] (*n* = 1) and Trail Making Test-5: Fine motor speed (TMT-5) [37] (*n* = 1). Seven articles (54%) reported adjustments for covariates and five (38%) articles reported proportion of motor difficulties based on a cut-off (Table 1). Two of the latter reported p-values for the corresponding motor test [21, 38], while three did not report p-values or reported continuous test scores for another test than was used for the dichotomized outcome [17, 23, 39].

**Table 1:** Characteristics of the included articles in order of participants’ age at the time of assessment

Citation	Setting	Design	SGA criteria	Birth year	Age	Motor outcome	Continuous scores	Adjustment for covariates	Cut-off for motor difficulties
Figueras et al. 2009 [40]	Spain	Cohort	BW < 10p	2006–2008	40 weeks	NBAS	Mean (SD)	Pregnancy factors, birth and neonatal factors, family and socioeconomic factors, sex and age	No
Campos et al. 2008 [22]	Brazil	Cohort	BW < 10p	2000–2003	1 month 2 months 3 months 6 months	BSID-II	Mean (SD)	No	No
Paulsen et al. 2023 [21]	Norway	Cohort	BW < 10p	2018–2019	3–7 months	AIMS, HINE, GMA MOS-R, TIMP	Mean (95% CI)^a^	Age	AIMS < 10p, HINE < 10p, GMA MOS-R < 25, TIMP <-0.5SD
Mello et al. 2014 [41]	Brazil	Cohort	BW < 10p	2000–2001	1 year	BSID-II	Mean (SD)	No	No
Evensen et al. 2009 [23]	Norway	Cohort	BW < 10p	1986–1988	1 year 5 years 14 years	BSID PDMS Movement ABC	Mean (SD)	No	BSID Motor < 2SD, PDMS < 5p Movement ABC < 5p
Juneja et al. 2005 [38]	India	Cohort	BW < 2000 g	2000–2001	18 months	BSID	Mean (SD)	No	BSID Motor < 85
Savchev et al. 2013 [18]	Spain	Cohort	BW < 10p	2007–2009	2 years	BSID-III	Mean (SD)	Birth and neonatal factors, family and socioeconomic factors and sex	No
Simões et al. 2015 [43]	Spain	Cohort	BW < 10p	n.r.	2 years	BSID-III	Mean (SD)^b^	Birth and neonatal factors, family and socioeconomic factors, sex and age	No
O’Neill et al. 2016 [44]	USA	Cohort	BW < 10p	2009–2011	2 years	BSID-III	Median (IQR)	Odds ratio adjusted for pregnancy factors, family and socioeconomic factors, sex	No
Sommerfelt et al. 2002 [17]	Norway and Sweden	Cohort	BW < 15p	1986–1988	5 years	GP, PDMS	Mean (SD)	No	PDMS < 5p
Klarić et al. 2013 [39]	Croatia	Cohort	BW < 10p	2002–2004	6 years	Touwen neurological examination	Median (IQR)	No	Developmental Coordination Disorder
Emond et al. 2006 [42]	Brazil	Cohort	BW 1500–2499 g	1993–1994	8 years	Movement ABC (3 items)	Mean (SD)	Birth and neonatal factors, family and socioeconomic factors, child factors at assessment	No
Weider et al. 2022 [24]	Norway	Cohort	BW < 10p	1986–1988	26 years	GP, TMT-5	Mean (SD)	Family and socioeconomic factors, sex	No

AIMS: Alberta Infant Motor Scale, BSID: Bayley Scales of Infant Development, BSID-II: Bayley Scales of Infant Development – Second edition, BSID-III: Bayley Scales of Infant and Toddler Development – Third edition, BW: Birth weight, CI: Confidence interval, GMA MOS-R: General Movements Assessment Motor Optimality Score – Revised, HINE: Hammersmith Infant Neurological Examination, IQR: Interquartile range, Movement ABC: Movement Assessment Battery for Children, NBAS: Neonatal Behavioral Assessment Scale, n.r.: Not reported, p: Percentile, PDMS: Peabody Developmental Motor Scales, SD: Standard deviation, SGA: Small for gestational age, TIMP: Test of Infant Motor Performance, TMT-5: Trail Making Test-5: Fine motor speed

^a^Adjusted for age at assessment

^b^Adjusted for gestational age, maternal low economic status, breast-feeding > 4 months, sex and age at assessment

### Quality of included studies

Quality scores ranged from 7 to 9 with a mean score of 8.2 (SD 0.8) for the included cohort studies, based on the Newcastle-Ottawa Scale [19]. All studies were rated high on representativeness, selection of the non-exposed cohort, demonstration that outcome was not present at start of the study and enough follow-up time for outcomes to occur, whereas adequacy of follow-up varied the most (Table 2).

**Table 2:** Quality assessment of the included studies

Citation	Representa-tiveness of the exposed cohort	Selection of the non-exposed cohort	Ascertain-ment of exposure	Demonstration that outcome of interest was not present at start of study	Comparability of studies on the basis of the design or analysis	Assessment of outcome	Follow-up long enough for outcomes to occur	Adequacy of follow up	Overall quality score
Figueras et al. 2009 [40]	A*	A*	A*	A*	A**	A*	A*	B*	9
Paulsen et al. 2023 [21]	A*	A*	A*	A*	A**	A*	A*	B*	9
Campos et al. 2008 [22]	A*	A*	A*	A*	A*	A*	A*	D	7
Mello et al. 2014 [41]	A*	A*	A*	A*	A*	A*	A*	D	7
Evensen et al. 2009 [23]	A*	A*	A*	A*	B*	A*	A*	B*	8
Juneja et al. 2005 [38]	A*	A*	A*	A*	A**	A*	A*	D	8
Savchev et al. 2013 [18]	A*	A*	A*	A*	A**	A*	A*	D	8
Simões et al. 2015 [43]	A*	A*	A*	A*	A**	A*	A*	B*	9
O’Neill et al. 2016 [44]	A*	A*	A*	A*	A**	A*	A*	B*	9
Sommerfelt et al. 2002 [17]	A*	A*	B*	A*	A**	A*	A*	B*	9
Klarić et al. 2013 [39]	A*	A*	B*	A*	A**	D	A*	C	7
Emond et al. 2006 [42]	A*	A*	A*	A*	A**	A*	A*	C	8
Weider et al. 2022 [24]	A*	A*	A*	A*	A**	A*	A*	B*	9

### Scores on standardized motor tests

Table 3 shows scores on standardized motor tests in the SGA and control groups as reported in the included articles. Seven (54%) articles reported poorer motor scores in individuals born SGA compared with controls [17, 18, 22, 39–42], while five (38%) articles reported no group differences [21, 24, 38, 43, 44], and one article did not report p-values for the group comparisons [23].

**Table 3:** Scores on standardized motor tests in the SGA and control groups as reported in the included articles

Citation	SGA, *n*	Control, *n*	Age, mean (SD)	SGA group		Control group		*P*-value	Difference in mean/median	Difference in SD
				**NBAS; Mean (SD)**						
Figueras et al. 2009 [40]	102	100	40 (1) weeks	5.15	(0.8)	5.67	(0.8)	< 0.0001	-0.52	-0.65
				**AIMS; Mean (95% CI)**						
Paulsen et al. 2023 [21]	34	163	3–7 months	18.1	(17.0–19.3)^a^	17.9	(17.4–18.4)^a^	n.s.	0.2	
				**GMA MOS-R; Mean (95% CI)**						
Paulsen et al. 2023 [21]	28	86	3–7 months	24.5	(23.6–25.4)	25.4	(24.9–5.8)	n.s.	-0.9	
				**HINE; Mean (95% CI)**						
Paulsen et al. 2023 [21]	39	168	3–7 months	62.5	(61.1–63.8)^a^	63.3	(62.6–63.9)^a^	n.s.	-0.8	
				**TIMP; Mean (95% CI)**						
Paulsen et al. 2023 [21]	18	54	3–7 months	113.8	(109.4–118.1)^a^	115.2	(112.7–17.8)^a^	n.s.	-1.4	
				**BSID-II; Mean (SD)**						
Campos et al. 2008 [22]	18	45	1 month	93.5	(7.89)	93.96	(7.39)	0.994	-0.46	-0.06
	25	43	2 months	89.76	(6.12)	93.49	(7.58)	0.008	-3.49	-0.46
	22	46	3 months	81.45	(7.27)	84.74	(9.20)	0.147	-3.20	-0.35
	24	42	6 months	88.54	(8.22)	93.31	(9.11)	0.038	-4.77	-0.36
Mello et al. 2014 [41]	22	47	12 months	91.14	(15.34)	98.79	(13.24)	0.047	-7.65	-0.58
				**BSID; Mean (SD)**						
Evensen et al. 2009 [23]	45	71	13.3 (0.6) months	104.6	(13.0)	108.9	(12.0)	n.r.	-4.3	-0.36
Juneja et al. 2005 [38]	50	30	18 (2) months	93.2	(19.7)	99.5	(10.3)	0.09	-6.3	-0.61
				**BSID-III; Mean (SD)**						
Savchev et al. 2013 [18]	112	111	24.0 (1.9) months	94.2	(13.1)	100.0	(14.4)	0.002	-5.8	-0.40
Simões et al. 2015 [43]	33	26	23 (1.5) months	97.1	(13.0)^b^	104.9	(15.5)^b^	0.091	-7.8	-0.50
				**BSID-III; Median (IQR)**						
O’Neill et al. 2016 [44]	51	189	24 months, 0 days – 30 months, 29 days	103	(94–110)	103	(97–110)	0.95	0	0.08^c^
				**PDMS; Mean (SD)**						
Evensen et al. 2009 [23]	49	73	5.3 (0.3) years^d^	79.1	(5.3)	80.7	(3.3)	n.r.	-1.6	-0.48
				58.7	(4.8)	59.1	(4.3)	n.r.	-0.4	-0.09
				105.2	(8.1)	105.9	(4.2)	n.r.	-0.7	-0.17
				**Touwen neurological examination; Median (range)**						
Klarić et al. 2013 [39]	50	50	5 years, 6 months – 7 years^e^	104.5	(66–168)	100	(72–134)	0.861	4.5	
				11	(2–35)	3	(1–9)	< 0.001	8	
				24	(17–29	29	(21–29)	< 0.001	-5	
				32	(22–50)	24	(22–34)	< 0.001	8	
				13.5	(2–24)	2	(0–8)	< 0.001	11.5	
				0	(0–4)	0	(0–2)	0.012	0	
				32.5	(20–39)	40	(25–42)	< 0.001	7.5	
				11	(0–24)	0	(0–10)	< 0.001	11	
				16	(2–24)	4	(0–10)	< 0.001	12	
				0	(0–4)	0	(0–1)	0.012	0	
				5	(0–12)	0	(0–4)	< 0.001	5	
				**Movement ABC; Mean (SD)**						
Emond et al. 2006 [42]	83	81	8 years^f^	18.0	(6.5)	17.9	(4.6)	0.51	0.1	0.02
	71	78		6.2	(2.0)	7.0	(1.9)	0.02	-0.8	-0.42
	83	81		10.9	(6.2)	12.9	(5.4)	0.03	-2.0	-0.37
Evensen et al. 2009 [23]	57	77	14.2 (0.3) years^g^	7.1	(5.5)	6.2	(4.2)	n.r.	0.9	0.21
				2.1	(3.1)	1.2	(1.6)	n.r.	0.9	0.56
				1.3	(1.5)	1.6	(1.9)	n.r.	-0.3	-0.16
				3.7	(3.0)	3.4	(2.8)	n.r.	0.3	0.11
				**GP; Mean (SD)**						
Sommerfelt et al. 2002 [17]	311	321	5 years	49.2	(11.2)	50.8	(8.6)	0.04	-1.7	-0.19
Weider et al. 2022 [24]	63	81	26.5 (0.5) years	D: 61.9	(10.4)	58.4	(8.6)	0.174	3.5	0.41
				ND: 68.8	(9.5)	63.8	(9.0)	0.147	5.0	0.56
				**TMT-5; Mean (SD)**						
Weider et al. 2022 [24]	63	81	26.5 (0.5) years	22.3	(5.7)	21.4	(5.6)	0.412	0.9	0.16

AIMS: Alberta Infant Motor Scale, BSID: Bayley Scales of Infant Development, BSID-II: Bayley Scales of Infant Development – Second edition, BSID-III: Bayley Scales of Infant and Toddler Development – Third edition, CI: Confidence interval, D: Dominant hand, GMA MOS-R: General Movements Assessment Motor Optimality Score – Revised, GP: Grooved Pegboard, HINE: Hammersmith Infant Neurological Examination, IQR: Interquartile range, Movement ABC: Movement Assessment Battery for Children, NBAS: Neonatal Behavioral Assessment Scale, ND: Non-dominant hand, n.r.: Not reported, n.s.: Not significant, PDMS: Peabody Developmental Motor Scales, SD: Standard deviation, SGA: Small for gestational age, TIMP: Test of Infant Motor Performance, TMT-5: Trail Making Test-5: Fine motor speed

Lower scores on AIMS, BSID, BSID-II, BSID-III, GMA MOS-R, HINE, Movement ABC item scores, NBAS, TIMP, Touwen neurological examination and PDMS indicate more difficulties

Higher scores on GP, Movement ABC total score and subscores, and TMT-5 indicate more difficulties

^a^Adjusted for age at assessment

^b^Adjusted for sex, maternal low economic status, gestational age, breast-feeding > 4 months, and age at assessment

^c^Hedges g effect size as reported in original article

^d^Subscores of eye-hand coordination, balance and locomotor

^e^Scores of sensorimotor function, posture, balance, coordination, fine motor skills, dyskinesia, muscle power, quantity of movements, associated movements, visual function and quality of movements

^f^Items of manual dexterity (treading beads onto a lace), eye-hand coordination (throwing a bean bag into a box) and dynamic balance (heel-toe walking)

^g^Movement ABC total score and subscores of manual dexterity, ball skills and static/dynamic balance

**Fig. 2:**
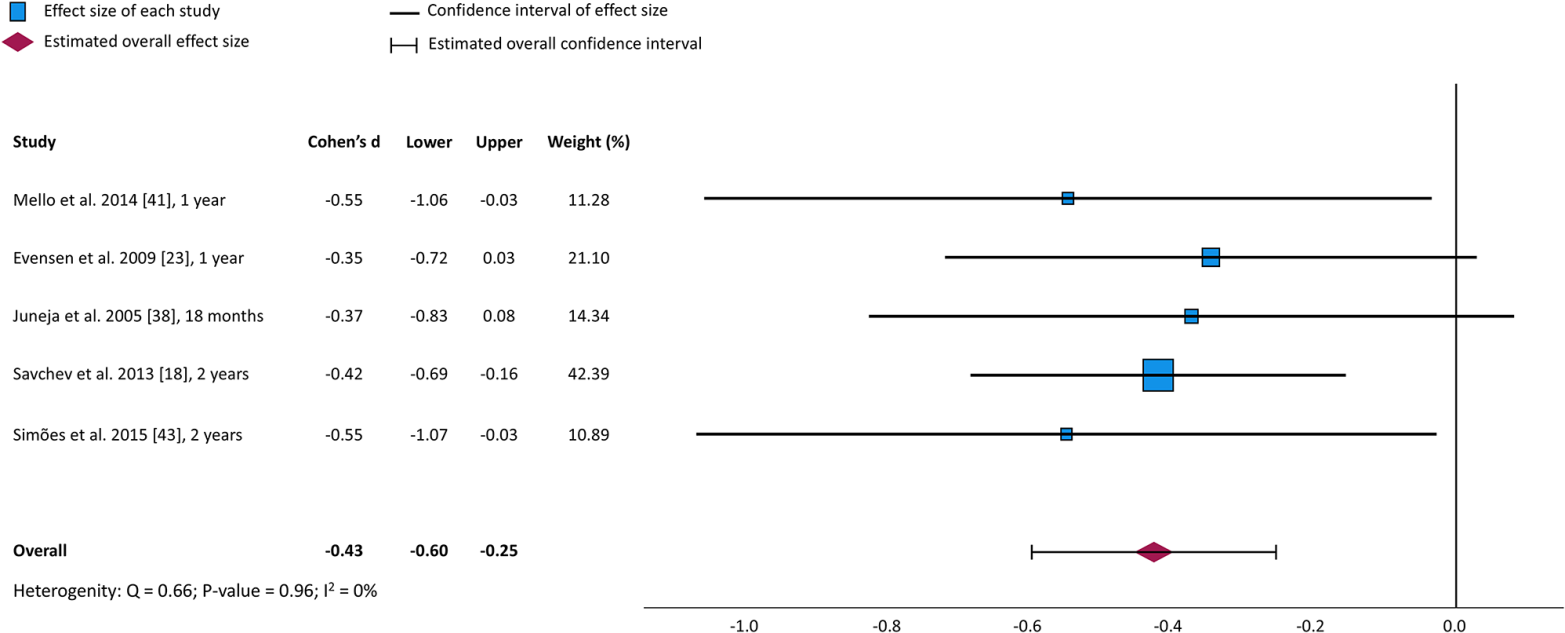
Effect sizes and heterogeneity statistics for scores on the Bayley Scales of Infant Development, in order of participants’ age at time of assessment

The NBAS was used in one study at 40 weeks of age and yielded unadjusted differences in mean scores of -0.52 points [40], corresponding to 0.65 SD units lower than in controls. Among the seven articles reporting results from the BSID, BSID-II or BSID-III, unadjusted mean scores were 3.49 to 7.65 points lower in children born SGA than in controls at 2, 6 [22] and 12 months [41], and at 2 years [18], corresponding to differences of -0.40 to -0.58 SD units. One article reported similar unadjusted mean scores in the SGA and control group at 1 and 3 months [22] and two articles reported no statistically significant group differences in mean scores at 18 months [38] or in median scores at 2 years [44]. The overall effect size from five studies reporting mean (SD) of the BSID, BSID-II or BSID-III at 1 to 2 years of age [18, 23, 38, 41, 43] was -0.43 (95% CI: -0.60 to -0.25) (Fig. 2) and data were homogenously distributed (*p* = 0.096).

In 5-year-old children, unadjusted PDMS scores were 0.7 to 1.6 points lower in the SGA group than in the control group [23], corresponding to differences of 0.17 to 0.48 SD units, although p-values were not reported. In a larger sample, Sommerfelt et al. [17] reported a 1.7 points difference in mean GP scores, corresponding to 0.19 SD units. Klarić et al. [39] applied Touwen neurological examination in children with asymmetrical IUGR at 6 years of age and found differences in median scores ranging from 5 points for balance to 12 points for associated movements, while sensorimotor function did not differ. Movement ABC total score was 0.9 points poorer, corresponding to 0.21 SD units in 14-year-old children [23]. Differences in manual dexterity ranged from 0.1 to 0.9 (0.02 to 0.56 SD units), ball skills from 0.3 to 0.8 (0.16 to 0.42 SD units) and balance from 0.3 to 2.0 (0.11 to 0.37 SD units) in 8- and 14-year-old children [23, 42]. The only adult study found no significant group differences in TMT-5 or GP mean scores [24].

Of the seven articles reporting adjustments for covariates, two studies adjusted for pregnancy factors, four studies adjusted for birth and neonatal factors, six studies adjusted for family and socioeconomic factors, five studies adjusted for sex, two studies adjusted for age, and one study adjusted for child factors at assessment. Adjusted for age at time of assessment, Paulsen et al. [21] found no significant group differences using the AIMS, GMA MOS-R, HINE and TIMP in 3–7 months old infants. Simões et al. [43] found a difference of -7.8 points on the BSID-III at 2 years of age, corresponding to -0.50 SD units, adjusted for gestational age, maternal economic status, breastfeeding > 4 months, sex and age at assessment. In studies reporting both unadjusted and adjusted results, adjustment for different covariates such as pregnancy factors, birth and neonatal factors, family and socioeconomic factors, child factors at assessment, sex and age did not affect the motor outcomes [18, 24, 40, 42]. Using odds ratio (OR) as estimate of the risk for BSID-III Motor scores < 85 points, O’Neill et al. [44] found no difference between crude OR and OR adjusted for pregnancy factors, family and socioeconomic factors and sex in the SGA group at 2 years of age.

### Proportions of motor difficulties

**Fig. 3:**
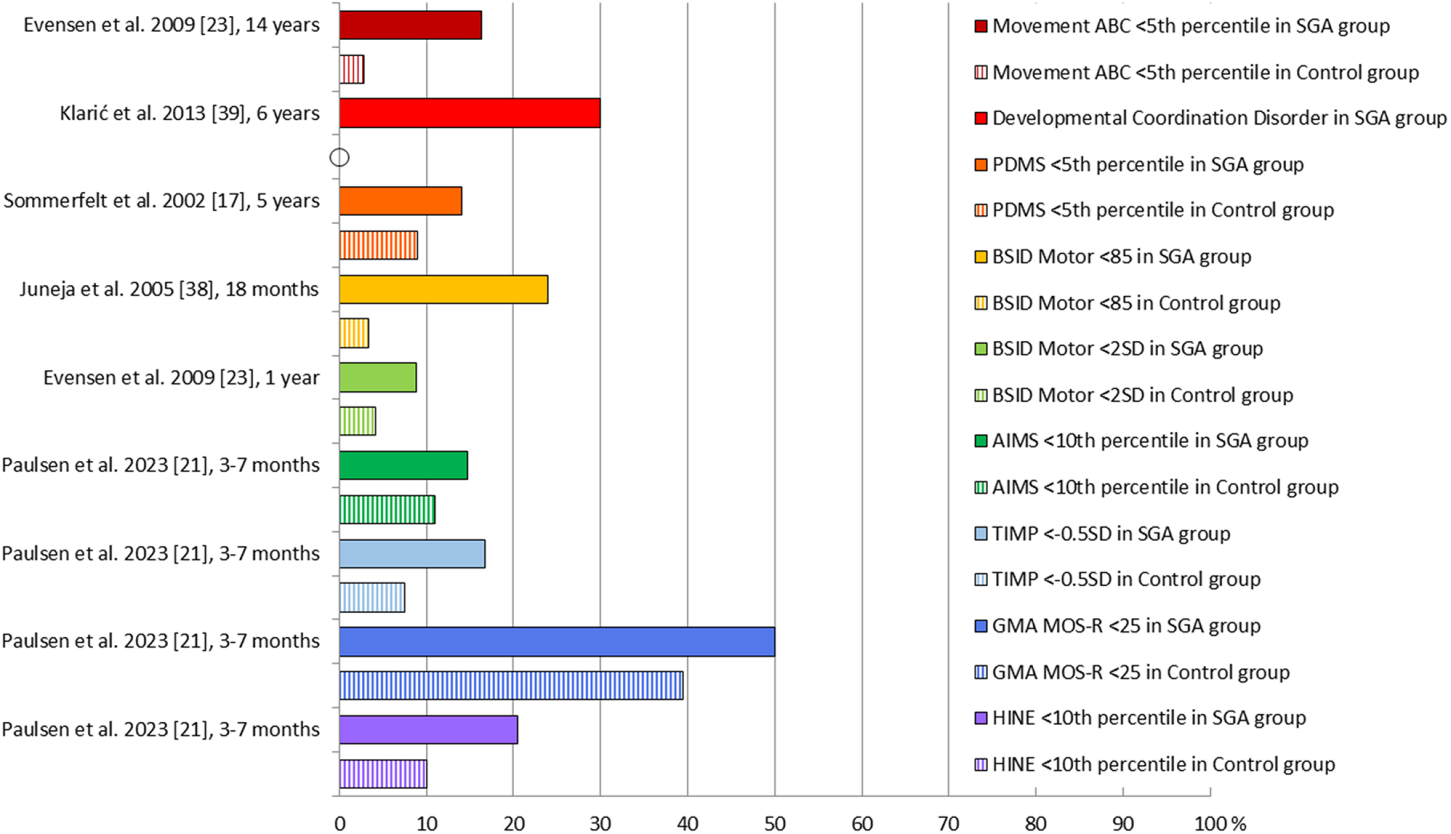
Proportions of children with motor difficulties in the SGA (solid bars) and control groups (vertical stripes). AIMS: Alberta Infant Motor Scale, BSID: Bayley Scales of Infant Development, GMA MOS-R: General Movements Assessment Motor Optimality Score – Revised, HINE: Hammersmith Infant Neurological Examination, Movement ABC: Movement Assessment Battery for Children, PDMS: Peabody Developmental Motor Scales, SD: Standard deviation, SGA: Small for gestational age, TIMP: Test of Infant Motor Performance

The five articles reporting proportions of motor difficulties are shown in Fig. 3. Although not statistically significant, the proportion of children with HINE scores < 10th percentile in the SGA group was twice as large as for the control group at 3–7 months, with 20.5% versus 10.1% [21]. The proportion of children with GMA MOS-R < 25 points was 50% in the SGA group versus 39.5% in the control group [21]. Also, the proportions of children with AIMS scores < 10th percentile and TIMP scores <-0.5 SD were higher in the SGA group compared with the control group at this age [21].

At 1 year, the proportion of children with BSID Motor scores < 2 SD was 8.9% in the SGA group and 4.2% in the control group, although no p-value was reported [23]. At 18 months, the proportion of children with BSID Motor scores < 85 points was 24.0% in the SGA group and 3.3% in the control group, with an OR of 9.16 (95% CI: 1.12 to 199.25), *p* = 0.03 [38]. The proportion of children with PDMS scores < 5th percentile at 5 years was higher in the SGA group with 14% versus 9% in the control group, OR 1.7 (95% CI: 1.0 to 2.7), *p* = 0.047 [17]. At 6 years, Klarić et al. [39] reported that 30% of the children with asymmetrical IUGR and none in the control group were diagnosed with Developmental Coordination Disorder. At 14 years, the proportion of children with Movement ABC scores < 5th percentile was more than five times higher in the SGA group, with 16.3% versus 2.8% in the control group [23].

## Discussion

### Main findings

In this systematic review on motor outcomes in children and adults born SGA at term, 13 original articles that fulfilled the inclusion criteria were included in a qualitative synthesis and five (38%) were included in a meta-analysis. In total, nine studies were from high-income countries. The studies had mainly assessed motor outcomes in early childhood, and only one study had examined adults born SGA at term. Seven (54%) articles reported poorer scores on standardized motor tests in individuals born SGA compared with controls, while five (38%) articles reported no group differences, and one article did not report p-values for the group comparisons. The pooled effect size in young childhood was moderate. Adjustments for covariates did not change the results. Five (38%) articles reported proportions of motor difficulties that ranged from 8.9 to 50% in the SGA group from childhood to adolescence.

### Strengths and limitations

The current systematic review contributes to the evidence regarding motor outcomes in individuals born SGA at term. Strengths of this study include the systematic literature search in two databases. Screening and quality assessment were performed by independent investigators, and the quality of the included studies was high. We included studies from both high- and middle-income countries, thus our findings may be generalizable to a large part of the world. Most studies used the 10th percentile definition of SGA, and we do not know if all individuals had suffered from FGR. We only included studies using standardized motor tests. Seven of the studies used a version of the BSID in the first two years of life, five of which were included in a meta-analysis. We did not search for studies using neurological tests, as we primarily focused on motor tests, even though neurological tests may include motor components. However, neurological tests also assess other components of neurological functioning, and some children may have neurological impairments but perform well on motor tests. Only one study assessed motor skills in adulthood, thus there is limited evidence regarding motor skills at older ages. However, in early childhood our findings were confirmed by the meta-analysis.

### Consistency with literature

In the seven articles reporting lower scores on standardized motor tests in the SGA group, effect sizes were small to moderate (0.19–0.65 SD units). However, five articles did not report significant group differences. This discrepancy seemed not related to setting, age at assessment nor type of motor assessment. However, small sample sizes may have played a role. In two of the articles not reporting significant differences, effect sizes were in the range of -0.35 [22] to -0.50 [43], which could be significant in larger study samples. In the meta-analysis of five studies using a version of the BSID between 1 and 2 years of age, the pooled effect size was -0.43 (95% CI: -0.60 to -0.25). Our results are consistent with other systematic reviews [13–15]. Even though they did not focus solely on motor skills or include only individuals born SGA at term, they also demonstrated lower neurodevelopmental scores during childhood. In 20 studies of children born after 35 weeks of gestation with IUGR identified *in utero*, Murray et al. [14] found lower motor scores from 1 month to 12 years of age corresponding to -0.34 SD difference from controls. Arcangeli et al. [13] included 29 studies of neurodevelopment in children up to 10 years of age who were born SGA and/or with FGR at term. They found standardized neurodevelopmental scores that were 0.31–0.32 SD below the scores of controls. Meher et al. [15] concluded that children born SGA with cerebral redistribution were at higher risk for poorer neurodevelopmental outcomes in early childhood up to 2 years of age. The conclusion was based on comparing NBAS and BSID scores from three studies including term-born children and two studies including both term and preterm born children. None of the previous reviews reported effect of sex. While male sex is reported to be associated with poorer motor skills in the general population [45], this has not been found in children born preterm [45] and our findings did not support this association in children and adults born SGA. Rather, adjustment for sex in five of the included articles did not change the results.

### Biological plausibility

As FGR is a compensational mechanism caused by maternal, placental, fetal or genetic factors [3], compromised growth and reduced energy reserve could have increased the risk of poor motor outcomes. Furthermore, developmental delay is more likely in children of lower-income families [38] and being born SGA is also more frequent in mothers with lower body weight and from low socioeconomic level [40]. However, adjustments for potential confounders did not change the results in the included studies. Still, we found a discrepancy in results between studies. This may be due to different populations, sample sizes and birth weight standards used, which may result in different proportions of infants suffering from FGR and genetically small infants.

### Clinical implications

Motor skills are essential for children and necessary to master practical tasks in everyday living. Consequences of motor difficulties may thus become apparent in the daily life of children and adolescents, and have implications for social functioning and peer relations [46]. In early childhood, developing motor control provides infants with opportunities for interacting with the social environment as well as their physical surroundings, and therefore can be viewed as part of an interactive developmental process including perceptual, social, and cognitive abilities [46]. Motor difficulties may also potentially affect physical activity and health-related quality of life. Studies of adults born SGA at term have revealed a higher prevalence of psychiatric disorders and mental health problems [47, 48]. However, in a recent study using accelerometers and self-report, they were not less physically active compared with controls [49]. Nevertheless, there are few studies on outcomes of being born SGA in adult life. More research is needed to examine potential long-term consequences of the poorer motor outcomes found in this systematic review. In any case, being born SGA may be a reason to initiate early assessment to identify motor difficulties and provide intervention or follow-up if needed.

## Conclusions

In conclusion, this systematic review shows that being born SGA, also at term, may be a risk factor for poorer motor outcomes throughout childhood, confirmed by a meta-analysis in early childhood. The majority of included studies reported lower scores on standardized motor tests and higher proportions of motor difficulties from early childhood to adulthood, although published studies in later childhood and adulthood were few. Further research is needed to establish the risk of adult motor difficulties and related consequences in individuals born SGA at term.

## Electronic supplementary material

Below is the link to the electronic supplementary material.

Supplementary Material 1: Table S1. Literature search in PubMed and Embase, last updated October 9, 2023.

Supplementary Material 2: Table S2. Newcastle-Ottawa criteria for the present systematic review.

Supplementary Material 3: Table S3. Standardized motor tests used in the included articles.

## Data Availability

All data generated or analyzed during this study are included in this published article [Table 3; Fig. 3].

## Abbreviations

AGA: Appropriate for gestational age
AIMS: Alberta Infant Motor Scale
BSID: Bayley Scales of Infant Development
BSID-II: Bayley Scales of Infant Development – Second Edition
BSID-III: Bayley Scales of Infant and Toddler Development – Third Edition
BW: Birth weight
CI: Confidence interval
GMA MOS-R: General Movements Assessment Motor Optimality Score – Revised
FGR: Fetal growth restriction
GP: Grooved Pegboard
IQR: Interquartile range
IUGR: Intrauterine growth restriction
Movement ABC: Movement Assessment Battery for Children
NBAS: Neonatal Behavioral Assessment Scale
PDMS: Peabody Developmental Motor Scales
SD: Standard deviation
SGA: Small for gestational age
TIMP: Test of Infant Motor Performance
TMT-5: Trail Making Test-5: Fine motor speed
