# Supplementary material for: Motor outcomes in individuals born small for gestational age at term: a systematic review
Source: BMC Pediatr. 2024 Nov 11;24:718. doi: 10.1186/s12887-024-05187-y (PMC11552374; doi:10.1186/s12887-024-05187-y)
Supplement: Supplementary file 1 — Supplementary Material 1: Table S1. Literature search in PubMed and Embase, last updated October 9, 2023. [file 12887_2024_5187_MOESM1_ESM.docx]

**Table S1.** Literature search in PubMed and Embase, last updated October 9, 2023.

| **Search number** | **Search terms** | **Number of hits** |
| --- | --- | --- |
| **PubMed** | | |
| #1 | "Infant, Small for Gestational Age"[Mesh] OR "Fetal Growth Retardation"[Mesh] OR "Infant, Low Birth Weight"[Mesh] OR "small for gestational age" [tw] OR "intrauterine growth restriction" [tw] OR "fetal growth restriction" [tw] OR "fetal growth retardation" [tw] OR "infant low birth weight" [tw] OR "infant small for gestational age" [tw] OR "SGA" [tw] OR "Dysmatur*" [tw] OR "Small for age" [tw] OR "Small at term*" [tw] OR "Small at birth" [tw] | 70,765 |
| #2 | ((“Motor skill*”[tw] OR “motor function*”[tw] OR “motor impairment*”[tw] OR “motor delay*”[tw] OR “motor defici*”[tw] OR “motor problem*”[tw] OR “motor disorder*”[tw] OR “motor disturb*”[tw] OR “motor abilit*”[tw] OR “motor capabilit*”[tw] OR “motor challeng*”[tw] OR “motor disabilit*”[tw] OR “motor dysfunction*”[tw] OR “motor speed” [tw] OR “motor disfunction*”[tw] OR “motor difficult*”[tw])  OR ("coordination skill*"[tw] OR "coordination function*"[tw] OR "coordination impairment*"[tw] OR "coordination delay*"[tw] OR "coordination defici*"[tw] OR "coordination problem*"[tw] OR "coordination disorder*"[tw] OR "coordination disturb*"[tw] OR "coordination abilit*"[tw] OR "coordination capabilit*"[tw] OR "coordination challeng*"[tw] OR "coordination disabilit*"[tw] OR "coordination dysfunction*"[tw] OR "coordination speed"[tw] OR "coordination difficult*"[tw])  OR ("Movement skill*"[tw] OR "movement function*"[tw] OR "movement impairment*"[tw] OR "movement delay*"[tw] OR "movement defici*"[tw] OR "movement problem*"[tw] OR "movement disorder*"[tw] OR "movement disturb*"[tw] OR "movement abilit*"[tw] OR "movement capabilit*"[tw] OR "movement challeng*"[tw] OR "movement disabilit*"[tw] OR "movement dysfunction*"[tw] OR "movement speed"[tw])  OR ("balance skill*"[tw] OR "balance function*"[tw] OR "balance impairment*"[tw] OR "balance defici*"[tw] OR "balance problem*"[tw] OR "balance disorder*"[tw] OR "balance disturb*"[tw] OR "balance abilit*"[tw] OR "balance capabilit*"[tw] OR "balance challeng*"[tw] OR "balance disabilit*"[tw] OR "balance dysfunction*"[tw] OR "balance speed"[tw] OR "balance difficult*"[tw])  OR ("Motor Skills Disorders"[Mesh] OR "Motor Skills"[Mesh] OR “motor performance*”[tw] OR “motor skill disorder*”[tw] OR “developmental coordination disorder*”[tw] OR “DCD”[tw] OR “fine motor*”[tw] OR “gross motor*” [tw] OR “manual dexterit*”[tw] OR “hand function*”[tw] “ball skill*”[tw] OR “static balance*”[tw] OR “dynamic balance*”[tw] OR “postural control*”[tw] OR “postural stabilit*”[tw] OR “Alberta Infant Motor Scale*”[tw] OR “Bayley Scales of Infant Development*”[tw] OR “BSID”[tw] OR “Peabody Developmental Motor Scale*”[tw] OR “PDMS”[tw] OR “Movement Assessment Battery for Children”[tw] OR “Movement Assessment Battery for Children 2”[tw] OR “Movement ABC”[tw] OR “MABC”[tw] OR “MABC-2”[tw] OR “Bruininks-Oseretsky test of motor proficiency”[tw] OR “BOTMP”[tw] OR “BOT-2”[tw] OR 'motor dysfunction assessment'[tw])) | 170,893 |
| #3 | “Term Birth”[Mesh] OR “term birth*”[tw] OR “term-born*”[tw] OR “born at term”[tw] OR “term neonate*”[tw] OR “term newborn*”[tw] OR “full term*”[tw] OR “full-term*”[tw] OR “Term infant*” [tw] OR “Term bab*” [tw] | 41,522 |
| #4 | #1 AND #2 AND #3 | 186 |
| **Embase** | |  |
| #1 | "small for gestational age"/exp | 20,440 |
| #2 | “small for gestational age*”:ti,ab,kw | 18,335 |
| #3 | sga:ti,ab,kw | 17,214 |
| #4 | “intrauterine growth restriction”:ti,ab,kw | 11,444 |
| #5 | “intrauterine growth retardation”:ti,ab,kw | 8,029 |
| #6 | “fetal growth retardation”:ti,ab,kw | 2,331 |
| #7 | "low birth weight"/exp | 75,647 |
| #8 | “low birth weight”:ti,ab,kw | 43,359 |
| #9 | “infant low birth weight”:ti,ab,kw | 266 |
| #10 | “infant small for gestational age”:ti,ab,kw | 111 |
| #11 | "intrauterine growth retardation"/exp | 55,956 |
| #12 | #1 OR #2 OR #3 OR #4 OR #5 OR #6 OR #7 OR #8 OR #9 OR #10 OR #11 | 131,371 |
| #13 | "motor performance"/exp | 94,308 |
| #14 | "motor dysfunction"/exp | 969,261 |
| #15 | ((motor* OR movement OR coordinat* OR balance) NEAR/1 (ability* OR capability OR function* OR speed OR skill* OR fine OR gross OR disability* OR disfunction* OR dysfunction* OR difficult* OR disorder* OR disturbance* OR delay* OR deficit* OR impairment* OR problem* OR challeng*)):ti,ab,kw | 183,962 |
| #16 | "developmental coordination disorder"/exp | 1,979 |
| #17 | 'dcd':ti,ab,kw | 7,932 |
| #18 | “movement assessment battery for children”/exp | 696 |
| #19 | “movement assessment battery for children 2”/exp | 26 |
| #20 | “movement assessment battery for children”:ti,ab,kw | 890 |
| #21 | “movement abc”:ti,ab,kw | 236 |
| #22 | “m abc”:ti,ab,kw | 177 |
| #23 | mabc:ti,ab,kw | 809 |
| #24 | “m abc 2”:ti,ab,kw | 48 |
| #25 | “bruininks-oseretsky test of motor proficiency”/exp | 304 |
| #26 | “bruininks-oseretsky test of motor proficiency”:ti,ab,kw | 495 |
| #27 | botmp:ti,ab,kw | 97 |
| #28 | "bot 2":ti,ab,kw | 280 |
| #29 | “bayley scales of infant development”/exp | 3,624 |
| #30 | “bayley scales of infant development ii”/exp | 45 |
| #31 | bsid:ti,ab,kw | 1,141 |
| #32 | “peabody developmental motor scales 2”/exp | 11 |
| #33 | “peabody developmental motor scales”:ti,ab,kw | 277 |
| #34 | pdms:ti,ab,kw | 11,895 |
| #35 | “alberta infant motor scales”:ti,ab,kw | 33 |
| #36 | (aims NEAR/5 motor*):ti,ab,kw | 1,241 |
| #37 | "motor dysfunction assessment"/exp | 58,669 |
| #38 | #13 OR #14 OR #15 OR #16 OR #17 OR #18 OR #19 OR #20 OR #21 OR #22 OR #23 OR #24 OR #25 OR #26 OR #27 OR #28 OR #29 OR #30 OR #31 OR #32 OR #33 OR #34 OR #35 OR #36 OR #37 | 1,165,175 |
| #39 | "born at term":ti,ab,kw | 4,543 |
| #40 | “term-born”:ti,ab,kw | 2,488 |
| #41 | "term birth"/exp | 4,481 |
| #42 | “term birth”:ti,ab,kw | 3,108 |
| #43 | “term birth*”:ti,ab,kw | 4,535 |
| #44 | “term neonate*”:ti,ab,kw | 7,368 |
| #45 | “term newborn*”:ti,ab,kw | 7,288 |
| #46 | "full term*":ti,ab,kw | 26,437 |
| #47 | "term infant*":ti,ab,kw | 17,411 |
| #48 | "term bab*":ti,ab,kw | 2,747 |
| #49 | #39 OR #40 OR #41 OR #42 OR #43 OR #44 OR #45 OR #46 OR #47 OR #48 | 57,266 |
| #50 | #12 AND #38 AND #49 | 582 |
