# Supplementary material for: Motor outcomes in individuals born small for gestational age at term: a systematic review
Source: BMC Pediatr. 2024 Nov 11;24:718. doi: 10.1186/s12887-024-05187-y (PMC11552374; doi:10.1186/s12887-024-05187-y)
Supplement: Supplementary file 2 — Supplementary Material 2: Table S2. Newcastle-Ottawa criteria for the present systematic review. [file 12887_2024_5187_MOESM2_ESM.docx]

**Table S2.** Newcastle-Ottawa criteria for the present systematic review.

**NEWCASTLE - OTTAWA QUALITY ASSESSMENT SCALE FOR COHORT STUDIES**

Note: A study can be awarded a maximum of one star for each numbered item within the Selection and Outcome categories. A maximum of two stars can be given for Comparability

**Selection**

1) Representativeness of the exposed cohort

A) Truly representative of the average ***term-born SGA (not a sub-selection*** in the community*

B) Somewhat representative of the average ***term-born*** ***SGA*** in the community*

C) Selected group of users e.g. nurses, volunteers

D) No description of the derivation of the cohort

2) Selection of the non-exposed cohort

A) Drawn from the same community as the exposed cohort*

B) Drawn from a different source

C) No description of the derivation of the non-exposed cohort

3) Ascertainment of exposure

A) Secure record (e.g. surgical records)*

B) Structured interview*

C) Written self-report

D) No description

4) Demonstration that outcome of interest was not present at start of study ***(motor outcome was not known when the participants were recruited)***

A) Yes*

B) No

**Comparability**

1) Comparability of cohorts on the basis of the design or analysis

A) The study ***reports p-value for comparison of mean or median motor scores****

B) The study ***controls for* *covariates or gives*** ***proportion below cut-off for motor difficulties****

C) Cohorts are not comparable on basis of scores, covariates or proportions of motor difficulties

**Outcome**

1) Assessment of outcome

A) Independent blind assessment*

B) Record linkage*

C) Self-report

D) No description

2) Was follow-up long enough for outcomes to occur

A) Yes ***(after birth)****

B) No

3) Adequacy of follow up of cohorts

A) Complete follow up - all subjects accounted for*

B) Subjects lost to follow up unlikely to introduce bias > ***80%*** follow up ***rate up to 2 years or > 60% follow up rate beyond 2 years****

C) Follow up rate ***below criteria described in B)***

D) No statement
