# Supplementary material for: Motor outcomes in individuals born small for gestational age at term: a systematic review
Source: BMC Pediatr. 2024 Nov 11;24:718. doi: 10.1186/s12887-024-05187-y (PMC11552374; doi:10.1186/s12887-024-05187-y)
Supplement: Supplementary file 3 — Supplementary Material 3: Table S3. Standardized motor tests used in the included articles. [file 12887_2024_5187_MOESM3_ESM.docx]

**Table S3.** Standardized motor tests used in the included articles.

| Test | Age range | Items included in the test | Scoring |
| --- | --- | --- | --- |
| Alberta Infant Motor Scale (AIMS) [1] | 0-18 months | 58 items which assess the control and integrity of antigravity postures organized into four subscales: prone, supine, sitting and standing. | Scored as 1 point for ‘observed’ and 0 point for ‘not observed’. Infant’s age percentile is calculated by using the total score. |
| Bayley Scales of Infant Development (BSID) [2-4] | 0-42 months | 3 scales: cognitive, language (receptive and expressive communication subtests) and motor (fine and gross motor subtest). | Scaled scores with a mean score of 100 and a standard deviation of 15. Scores within 1 SD of the mean (85-115) are considered normal. |
| General Movements Assessment Motor Optimality Score – Revised  (GMA MOS-R) [5] | 3-5 months | 5 subcategories: temporal organization and quality of fidgety movements, quality of movement patterns, age adequate movement repertoire, postural patterns and movement character. | Summing scores of the five subcategories give a motor optimality score ranging from 5 to 28 points. Scores <25 are considered to be reduced. |
| Grooved Pegboard (GP) [6] | >5 years | Inserting 25 pegs, one at a time, into keyhole-shaped holes with various orientations in a 5×5 matrix. Dominant and non-dominant hand are tested. | Raw scores consist of time in seconds to complete the board and number of drops for each hand. Age-specific norms. |
| Hammersmith Infant Neurological Examination [7] | 3-24 months | 3 sections: neurological examination (26 items; cranial nerve function, posture, movements, tone and reflexes), observations of motor milestones and behavioral state. | Items in the neurological examination are scored from 0 to 3, with a maximum total score of 78 points. Scores ≤10^th^ percentile are regarded as suboptimal. |
| Neonatal Behavioral Assessment Scale (NBAS) [8] | 0-2 months | 35 items grouped in 6 clusters: habituation, motor, social-interactive, organization of state, regulation of state and autonomic nervous system. | Rated scores on a scale of 1 to 9 (9 being the best performance) except for 8 curvilinear scale items rescored as linear on a 5-, 6- or 8-point scale. |
| Movement Assessment Battery for Children (Movement ABC) [9] | 4-12 years | 8 items in 3 subscores; manual dexterity (3 items), ball skills (2 items), static/dynamic balance (3 items). | Raw scores converted to standard scores on a 6-point scale (0–5; 0 being optimal score) for each item and summarized to a total impairment score (maximum 40). Scores <5^th^ percentile indicate motor problems. |
| Peabody Developmental Motor Scale (PDMS) [10] | 0-83 months | Gross motor scale (170 items; reflexes, balance, receipt and propulsion, nonlocomotory and locomotor) and fine motor scale (112 items; grasping, hand use, eye-hand coordination and manual dexterity). | Items scored as:  0 = cannot perform item  1 = clear resemblance to correct performance  2 = correct performance |
| Test of Infant Motor Performance (TIMP) [11] | Birth (32 weeks)-4 months | 42 items (13 observed and 29 elicited): postural change, adaptation to handling, anti-gravity movement, visual reaction, auditory reaction and postural control of the head and body. | For observed items, score of 1 is given if present and 0 of absent. For elicited items scores vary from 1 to 6 points. Cut off value of -0.5 SD below the mean has high sensitivity for detecting problems in high-risk infants. |
| Touwen Neurological examination [12] | 0 months-walking | Cranial nerves, posture, tome, reflexes and reactions, trunk coordination and gross and fine motor functions. | Criteria for age-appropriate performance. |
| Trail Making Test-5: Fine motor speed (TMT-5) [13] | Adolescents and adults | Drawing a line as fast as possible in a directed order. | Scores are based on the time taken to complete the task. Longer times may suggest difficulties in visual motor skills, attention, or executive function. |

**References**

1. Piper M, Darrah J. *Motor assessment of the developing infant*. Philadelphia, PA: W.B. Saunders; 1994.
2. Bayley N. *Manual for the Bayley scales of infant development.* New York, NY: The Psychological Corporation; 1969.
3. Bayley N. *Bayley Scales on Infant Development* – *Second Edition: Manual.* San Antonio, TX: The Psychological Corporation; 1993.
4. Bayley N. *Bayley Scles of Infant and Toddler Development* – *Third Edition.* San Antonio, TX: Harcourt Assessment; 2006.
5. Einspieler C, et al., *Cerebral Palsy: Early Markers of Clinical Phenotype and Functional Outcome.* J Clin Med. 2019;8(10):1616.
6. *Lafayette Instrument Grooved Pegboard Test User Instructions.* Lafayette, IN: Lafayette Instrument Company, Inc.; 2002.
7. Haataja L, et al., *Optimality score for the neurologic examination of the infant at 12 and 18 months of age.* J Pediatr. 1999;135(2 Pt 1):153-61.
8. Nugent J, Brazelton T. *Preventive infant mental health: uses of the Brazelton scale*. In: Osofsky J, Fitzgerald H, editors. The Handbook of Infant Mental Health, Vol 2: Early Intervention, Evaluation and Assessment. New York, NY: Wiley; 2000, pp. 157–202.
9. Henderson S, Sugden D. *Movement Assessment Battery for Children. Manual.* London: The Psychological Corporation; 1992.
10. Folio M, Fewell R. *Peabody Developmental Motor Scales and Activity Cards. Manual.* Austin, TX: DLM Teaching Resources; 1983.
11. Campbell SK. *The test of Infant Motor Performance test user’s manual version 3.0 for the TIMP Version 5.* Chigaco, IL: Infant Motor Performance Scales, LLC; 2012.
12. Touwen B. *Examination of the Child With Minor Neurological Dysfunction. 2nd ed.* London: Spastics International Medical Publications; 1979.
13. Delis DC, Kaplan E, Kramer J. *Delis Kaplan Executive Function System*. San Antonio, TX: The Psychological Corporation; 2001.
